# Factors associated with acute depressive symptoms in patients with comorbid depression attending cardiac rehabilitation

**DOI:** 10.1186/s12872-018-0974-2

**Published:** 2018-12-10

**Authors:** Serdar Sever, Su Golder, Patrick Doherty

**Affiliations:** Department of Health Sciences, Faculty of Science, University of York, York, UK

**Keywords:** Cardiovascular disease, Cardiac rehabilitation, Depression, Depressive symptoms, History of depression, Comorbid depression, Observational study

## Abstract

**Background:**

The literature suggests that comorbid depression, defined in this paper as a history of depression prior to a cardiovascular event, has an impact on later onset depression as well as constituting increased risk of mortality and adverse cardiac events. However, which factors are associated with depression, specifically in patients with comorbid depression, is unclear. Therefore, this paper investigates the factors associated with depression in patients with comorbid depression attending cardiac rehabilitation (CR).

**Methods:**

This observational study used routinely collected data from the British Heart Foundation National Audit of Cardiac Rehabilitation for the time period between April 2012 and March 2017. CR participants with comorbid depression were selected as the study population. An independent t-test and chi-square test were used to compare the association between acute depression symptoms and baseline characteristics in this population.

**Results:**

A total of 2715 CR patients with comorbid depression were analysed. Characteristics associated with acute depressive symptoms in patients with comorbid depression were found to be: young age (MD: 2.71, 95% CI 1.91, 3.50), increased number of comorbidities (MD: -0.50, 95% CI -0.66, − 0.34), increased weight (MD: -1.94, 95% CI -3.35, − 0.52), high BMI (MD: -1.94, 95% CI -3.35, − 0.52), HADS anxiety (MD: -5.17, 95% CI -5.47, − 4.87), comorbid anxiety (52.4%, *p* <  0.001), physical inactivity (150 min moderate physical activity a week and 75 min vigorous exercise a week; 27.5%, *p* <  0.001; 5.6%, *p* <  0.001 respectively), smoking (12.7%, *p* <  0.001), and being less likely to be partnered (63.6%, *p* <  0.001).

**Conclusion:**

The study demonstrated the association between a variety of clinical and socio-demographic factors and depression. The findings of the research indicated that, at CR baseline assessment, caution must be taken with patients with comorbid depression, specifically those with higher level depressive symptoms at the start of rehabilitation. Furthermore, their multi-comorbid condition must also be taken into account. Patients with higher depression symptoms and comorbid depression scored five points higher on the HADS anxiety scale in comparison to patients with lower level depression symptoms at the start of CR, which demonstrated that anxiety and depression are interrelated and present together.

## Background

Cardiovascular disease (CVD) has a high mortality rate and was the primary cause of 17.7 million deaths worldwide in 2015 [1]. According to recent statistics from the European Society of Cardiology (ESC), 83.5 million people are living with CVD in 47 member countries [2]. Improving population CVD survival trends and an aging population have been associated with an increased number of comorbidities in patients with CVD, which represents a significant service delivery challenge [3]. Depression has been shown to be one of the most common comorbidities in CVD patients [4]. The prevalence of depression in cardiac patients varies according to the assessment methods used; for instance, a systematic review found that the percentage of myocardial infarction patients who met the Diagnostic and Statistical Manual of Mental Disorders (DSM-IV) clinical diagnosis tool for major depression was 15 to 20%, and this proportion increased when considering elevated depression symptoms [5]. Moreover, evidence has suggested that depression is an independent risk factor for the recurrence of cardiac events, cardiac related mortality [6, 7], and all-cause mortality [8–10]. In addition, a recent systematic review demonstrated that the combination of CVD and depression has a significant impact on service utilization and medical costs [11].

Cardiac rehabilitation is effective and recommended for CVD patients [12], and it also reduces depressive symptoms [13, 14]. Recent guidelines have emphasized that CR should be a multicomponent intervention that includes the management of psychosocial health [15, 16]. In order to assess and manage participants’ depression symptoms, an assessment of psychosocial health status is required. In UK CR, the Hospital Anxiety and Depression Scale (HADS) is used pre CR to tailor the intervention around patients’ needs and goals [17]. HADS is also a validated tool for screening depressive symptoms in cardiac patients in other countries [18–20]. Most research has focused on psychosocial health measures associated with the acute cardiac event, and little attention has been paid to comorbid depression [10]. One study showed that 50% of patients diagnosed with depression after MI had comorbid depression [21]. Other studies have found that heart failure patients with comorbid depression prior to the heart event were at higher risk of mortality and worse cardiac prognosis [22, 23]. Additionally, a meta-analysis revealed that CVD patients with comorbid depression and patients with depressive symptoms after the cardiac event were at increased risk of mortality and cardiac morbidity, whereas patients with comorbid depression only were at reduced risk [24]. Participant characteristics associated with higher level depressive symptoms in patients with comorbid depression may differ from those with comorbid depression who do not show depressive symptoms or the ones with low depression levels at baseline. Accordingly, the aim of this study is to determine the sociodemographic and clinical characteristics associated with acute depressive symptoms in patients with comorbid depression, in order to be able to better manage depression symptoms in patients with historic self-reported depression and tailor CR interventions. Furthermore, little research has been conducted into comorbid depression in patients attending CR, hence this study provides important insights from routine practice.

## Methods

The strengthening the reporting of observational studies in epidemiology (STROBE) checklist was used to report this study [25].

### Data collection

The analysis conducted was based on NACR data. The NACR monitors CR services and aims to improve the quality of CR programmes across the UK. Patient level data are collected daily by clinical programmes under Section 251 approval and input through a secure online portal hosted by NHS Digital. A link-anonymized version of baseline and post-CR assessments, carried out by programmes across the UK as part of routine practice, is transferred to the NACR at the University of York. The total number of services entering the data electronically is 224, which represents 74% of all programmes [17]. The data consist of patients who undergo CR in the UK, along with their demographics, initiating event, risk factors, treatment, medication, and outcomes.

### Participants

Based within the NACR data governance secure environment, data from the last five years — 1 April 2012 to 31 March 2017 — were extracted. Relevant guidelines have recommended the inclusion of patients with MI and heart failure and those who receive treatment for PCI and CABG in CR [26], which constituted the study population. During the study period, all the eligible patients (*N* = 2715) with comorbid depression who had pre and post-HADS assessments in CR were selected as participants.

### Measures

#### Comorbid depression

Comorbid depression was measured using a self-answered questionnaire provided by the NACR data which asked patients to identify whether they had ever been told by a doctor that they definitely had or had been treated for depression. It has been answered as ‘yes’ or ‘no’. CR practitioners did also verify whether patients have comorbid depression by case note review which increases the credibility of this variable. Therefore, comorbid depression is defined in this paper as depression history prior to heart event.

#### Comorbid anxiety

Comorbid anxiety was measured with another questionnaire, which patients answered if they had ever been told by a doctor that they definitely had or had been treated for anxiety. The answer options were ‘yes’ or ‘no’. Again CR practitioners did also confirm whether patients have comorbid anxiety by case note review. Thus, comorbid anxiety provided the measure of patients’ anxiety history prior to heart event.

#### Hospital anxiety and depression scale

The HADS is a screening tool which is used for measuring depressive symptoms in many clinical areas. It is a self-answered questionnaire; however, it is completed with the guidance of healthcare professionals when necessary. It is recommended that psychosocial health measurements (HADS) are assessed before and after CR, in order to tailor the intervention to the patient population [15]. The HADS score ranges from 0 to 21, where higher scores relate to worse symptoms, and has been found to be a reliable measure for the assessment of depression and anxiety symptoms in terms of internal consistency and test-retest reliability; therefore, it is recommended for use with cardiac patients [18–20]. HADS has 14 items in total, 7 item for anxiety symptoms and 7 for depression. Each item can get a score of 0 to 3 by which means minimum 0 and maximum 21 could be received for both anxiety and depression scores separately. HADS is licenced to NACR for use by services registered with NACR. In this study, CR baseline HADS depression measurement was used for the analysis and the clinical cut off point of 8 was used to categorize patients into low level depression (< 8) and higher level depression (≥ 8) groups [20]. The reason for this is a systematic review has shown that an optimal balance between sensitivity and specificity for HADS as a screening tool was often achieved at a cut of score of 8 for both HADS anxiety and HADS depression considering sensitivities and specificities for both scales roughly 0.80 [27]. Then, analyses were conducted comparing patients with HADS < 8 and HADS ≥8 in a subgroup of patients with comorbid depression. In addition, a comparison was conducted between the baseline HADS anxiety scores of the patients with low and high levels of depressive symptoms. To be clearer, pre CR HADS measurements both for anxiety and depression were used for the analyses in this study.

#### Total number of comorbidities

This variable determined the number of comorbidities present in each patient. A comparison was then conducted between the number of comorbidities in the higher and lower level depression groups. There are 19 different comorbidities in the NACR data, including hypertension, hypercholesterolemia, diabetes, angina, arthritis, osteoporosis, asthma, chronic bronchitis, and others.

#### Other variables

Age was used as a continuous variable in the analyses. Gender was categorised to male or female. Weight is measured in kilogrammes. BMI is calculated as weight in kilogrammes divided by squared height in meters and is used as a continuous variable in the analyses. Baseline, pre- CR, smoking measurements are categorised as to whether a patient is a current smoker or non-smoker. Alcohol intake is calculated by units of alcohol a patient consumed per week. One unit of alcohol is about equal to: half a pint of ordinary strength beer, lager or cider (3–4% alcohol by volume); or a small pub measure (25 ml) of spirits (40% alcohol by volume). Marital status is categorised into whether the patient is partnered or single. Baseline physical activity is measured by the question: ‘Do you take regular moderate physical activity of at least 30 minutes duration on average 5 times a week? (or its equivalent, 150 minutes over 7 days)’ and the response options were ‘yes’ or ‘no’. Moderate activity is explained in the questionnaire as anything that takes as much effort as a brisk walking or housework, carrying a light bag on level ground, mowing the lawn, painting and decorating and others. Another physical activity measurement question was ‘Do you do 75 minutes of vigorous exercise a week?’ Possible answers are again ‘yes’ or ‘no’. Vigorous activity is explained as anything that takes as much effort as running, vigorous swimming or cycling, an aerobics class, circuit training, digging in heavy ground, chopping wood or sports like football, rugby, squash or netball. All the variables included in this paper has been chosen in line with the literature and preliminary baseline assessments.

### Statistical analysis

The analyses were conducted using the IBM Statistical Package for social sciences software statistics version 24 (New York, USA). Patients with comorbid depression who had undertaken valid pre and post-HADS assessments were selected for the study population. The statistical significance level was set to 5%. Summary statistics were presented as mean and standard deviation unless otherwise stated. The mean difference between the high and low level depression groups for age, number of comorbidities, weight, BMI, weekly alcohol intake, and HADS anxiety were investigated using independent samples t-tests. The association between gender, comorbid anxiety, physical activity, smoking, marital status, and depression was investigated using chi-square tests. Finally, Cohen’s d effect size for continuous variables and phi effect size for categorical variables were reported.

### Ethics

The British Heart Foundation NACR data are managed by NHS Digital through a secure online platform where data are entered by approved members of clinical teams. NHS Digital detects the identifiable patient data which is then link-anonymized and transferred to the NACR team for data validation. Data on pre and post rehabilitation assessments and patient characteristics are collected for audit and service improvement purposes, which forms part of the NHS Digital contract. On this basis, no separate NHS ethical approval was required for the current study.

## Results

**Fig. 1:**
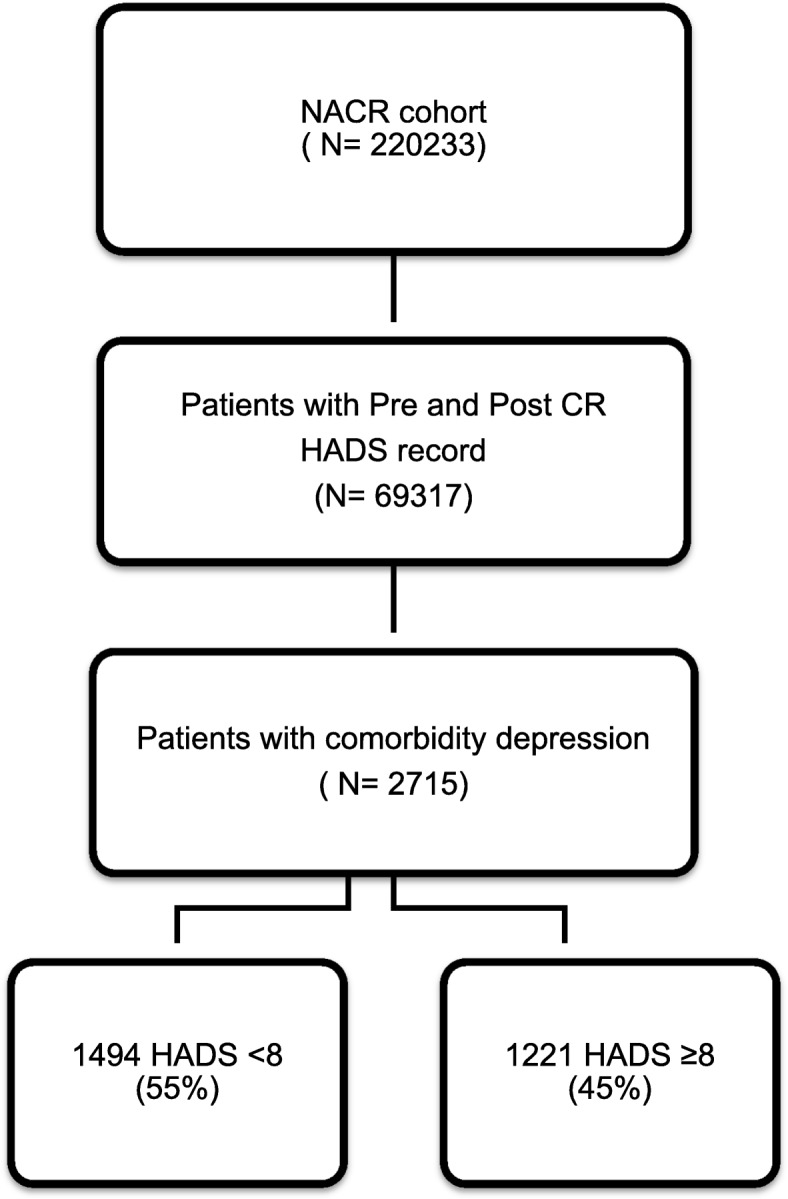
A flow diagram for study sampling

The study population included a total of 2715 participants with comorbid depression who had completed CR with valid pre and post HADS assessments. The flow diagram in Fig. 1 shows the total population during the study time period and the sample size of the study. Baseline characteristics in the context of HADS are presented in Table 1.

**Table 1:** Baseline characteristics with t-tests for the mean differences between high and low HADS depression groups

Variables	HADS < 8 group (*n* = 1494)	HADS ≥8 group (*n* = 1221)	Difference	95% CI	*P*	d
	Mean + SD	Mean + SD				
Age	63.49 ± 10.50	60.77 ± 10.53	2.71	1.91 to 3.50	< 0.001	0.26
Total Comorbidities	4.39 ± 2.10	4.90 ± 2.19	-0.50	-0.66 to −0.34	< 0.001	0.24
Weight	83.62 ± 17.04	85.56 ± 19.08	-1.94	-3.35 to −0.52	= 0.001	0.11
BMI	28.83 ± 5.14	29.54 ± 5.73	−0.71	−1.13 to − 0.28	< 0.001	0.13
Weekly Alcohol Intake	6.44 ± 10.67	5.66 ± 11.50	0.78	−0.12 to 1.70	0.09	0.07
HADS Anxiety Score Measurement	6.43 ± 3.80	11.60 ± 4.05	−5.17	− 5.47 to −4.87	< 0.001	1.32

*SD* standard deviation

An independent samples t-test was run to determine if there were differences in age, total number of comorbidities, weight, BMI, weekly alcohol intake, and HADS anxiety measurement between patients with low depression scores (HADS < 8) and higher depression (HADS) score. Participants with higher level depressive symptoms were younger, had a higher number of comorbidities, increased weight, higher BMI, and more anxiety symptoms than patients in the lower level depression group. However, the difference in alcohol intake was not statistically significant between both groups.

Chi-square tests for association between categories were conducted between gender, comorbid anxiety, 150 min physical activity a week, 75 min vigorous exercise a week, smoking, marital status, and depression symptoms. All the test variables other than gender were significantly associated with depression. Patients with comorbid depression and higher depressive symptoms at the start of CR were more likely to smoke, have comorbid anxiety, be physically inactive (both 150 min and 75 min), and single. The results of the chi-square tests are presented in Table 2.

**Table 2:** Results of the chi square tests for association

Variables	HADS < 8 Group %	HADS ≥8 Group %	*P*	Effect size
Female	33.6	33.6	0.99	0
Comorbid Anxiety (Yes)	41.8	52.4	< 0.001	0.11
150 min Physical Activity a Week (Yes)	43.6	27.5	< 0.001	0.17
75 min Vigorous Exercise a Week (Yes)	9.8	5.6	< 0.001	0.08
Smoking (Yes)	7.7	12.7	< 0.001	0.08
Partnered	71.5	63.6	< 0.001	0.08

## Discussion

Previous studies demonstrated the association of depressive symptoms with patient demographics and modifiable lifestyle risk factors. However, the extent of the association with comorbid depression was not clear. Thus, the current study investigated this association in patients with comorbid depression. The findings of the investigation revealed a variety of factors that were statistically significant in relation to acute depressive symptoms in patients with comorbid depression. In particular, depressive symptoms were associated with a higher number of comorbidities, increased weight, higher BMI, anxiety symptoms, comorbid anxiety, physical inactivity, and smoking in patients with historic comorbid depression. Additionally, patients with depression tended to be younger and single. However, no significant association was found for gender or alcohol intake.

The results are similar to those of previous studies which suggested that depressive symptoms are associated with reduced physical activity [28, 29], smoking [30–32], high BMI [33, 34], and increased weight [35, 36]. However, these studies often recruited patients with either new onset depression which occurred after a heart event or general population without CVD. Whereas this research also considered the comorbid depression population, which appears to be equally as important [22]. To our knowledge, the characteristics of participants with comorbid depression and those with higher level depressive symptoms after a cardiac incident have never been compared before to the patients with an absence of depressive symptoms after a heart event.

One finding was that BMI demonstrated a statistically significant difference of − 0.71 (95% CI, − 1.13 to − 0.28), *p* = 0.001. Although the BMI for both low level and high levels of depression groups is in same range by being overweight and the difference is less than 1 point, participants that are in the high levels of depressive symptoms group were close to the cut of point of 30 for the obese category with BMI: 29.54. Similarly, weight measurement had a significant difference of − 1.94 kilogrammes (95% CI, − 3.35 to − 0.52) between the higher level depression and lower level depression symptoms groups in participants with comorbid depression. Unlike prior studies, the total number of comorbidities was significantly higher in patients with higher level depressive symptoms, the mean difference being − 0.50 (95% CI, − 0.66 to − 0.34) [37]. The data included 19 different comorbidities and multi-comorbid patient populations in the analysis, which strengthens the importance of this finding. In addition, mean total number of comorbidities in NACR data is 1.60, whereas when we have factored in patients with comorbid depression this increased to above 4 which shows that patients with comorbid depression have higher total number of comorbidities. Besides, when patients with comorbid depression and also higher levels of depressive symptoms at the start of CR, they have higher total number of comorbidities than the ones with low levels of depressive symptoms.

This study confirmed that anxiety is associated with depressive symptoms. However, the mean HADS anxiety scores were significantly higher in more depressive patients compared to the lower level depression group patients with comorbid depression (− 5.17 — 95% CI -5.47 to − 4.87). This difference is relatively high given that HADS is scored out of 21 and this has not been seen in the most recent CR randomised controlled trial REACH HF and a feasibility randomised controlled trial WREN [38, 39]. In contrast to other studies, this paper investigated comorbid anxiety, which was prevalent in 52.4% of participants with higher level depressive symptoms, and this association was statistically significant. In general, therefore, it seems that both pre and post heart event anxiety is associated with depressive symptoms. Indeed, post heart event anxiety had the largest effect (d = 1.32, *p* <  0.001). Meanwhile, age and total number of comorbidities had small to moderate effect size, and this was relatively smaller for the rest of the variables.

The observed difference in weekly alcohol intake was not statistically significant between the high and low level depression groups participants, and in fact was less in participants with depression. This finding differs from previous research which has identified an association between increased alcohol consumption and depressive symptoms [40]. It is difficult to explain this result, but it might be related to the multi-comorbid conditions of the participants in this study, which may require a restriction in alcohol intake. Another clinically relevant finding was that physical inactivity and smoking were two modifiable cardiac risk factors associated with depressive symptoms. However, a bidirectional relationship may be present between physical activity and depression; thus, physical inactivity might have appeared as a consequence of depression. Although these results differ from one published study [41], they are consistent with the majority of others [28–32, 42].

Some patient demographics such as age and marital status were found to be significantly associated with higher depressive symptoms, whereby patients with higher level depression tended to be younger (MD = 2.71, 95% CI 1.91 to 3.50) and less likely to be partnered. These findings support previous research into the association between depressive symptoms, age [43, 44], and marital status [45]. This study was unable to demonstrate a gender difference in the context of females mentioned in the literature [34]. A possible explanation for this might be that the study sample included more females (33.6%), which may have affected the results. Although the mean age for the female participants was lower in the study sample (62.9 SD: 11.19 in comparison to 65.9 SD 8.9), gender did not demonstrate a significant association with higher level depressive symptoms. The data given above must be interpreted with caution, as the researchers included patients with comorbid depression to investigate whether patients with depressive symptoms have poorer baseline characteristics when they have a history of depression. Therefore, the study findings may not be generalizable to all CVD populations with higher level depressive symptoms. However, the findings for patient demographics in the study sample and all available participants with comorbid depression in the data were similar; for example, the mean age was 62 compared to 61, there were 33.6% females compared to 35%, and the proportion did not differ more than 5% for the other variables.

### Limitations

The sample was nationally representative of patients with comorbid depression in the UK. However, not all CR programmes in the UK provide complete patient records, and among the participants who had completed CR in the NACR data, 38% did not have a follow up assessment, which may have impacted sample representativeness [17, 46]. Additionally, this study was limited to CR participants, whose characteristics may differ from those who do not attend CR programmes [47]. The analysis of routinely collected patient data is useful for providing real-world understanding. The data used in this study captured participants with multi-comorbidities and more female participants than prior RCTs [12]. However, causal conclusions cannot be drawn through observational studies. The current study was limited to the variables available in the NACR, and there may be other variables associated with depressive symptoms which were not captured by the data. Another possible limitation is that comorbid depression was a self-reported measure, which might be prone to recall bias [48]. HADS was used for the measurement of anxiety and depression symptoms in this study and continues to be a validated measure in NACR and major research studies. However, a systematic review conducted in 2012 suggested that HADS was more a measure of emotional distress with poor differentiation between the constructs of anxiety and depression [49].

## Conclusion

The purpose of this study was to investigate the factors associated with depressive symptoms in patients with comorbid depression. Returning to the hypothesis defined at the beginning of this study, it is now possible to state that patients with comorbid depression and those with higher level depressive symptoms prior to CR tended to have a high number of comorbidities, high comorbid anxiety, increased weight, high BMI, anxiety symptoms, smoking, and lower levels of physical activity. In addition, they were younger and single. Thus, at baseline assessment, CR programmes should give special attention to patients with comorbid depression and those patients that have higher level depressive symptoms at the start of rehabilitation. Further investigation into comorbid depression and links between depressive symptoms and patient characteristics are strongly recommended.

## Abbreviations

BHF: British Heart Foundation
BMI: Body Mass Index
CABG: Coronary artery bypass graft
CR: Cardiac rehabilitation
CVD: Cardiovascular disease
ESC: European Society of Cardiology
HADS: Hospital Anxiety and Depression Scale
MI: Myocardial infarction
NACR: National Audit of Cardiac Rehabilitation
PCI: Percutaneous coronary intervention
